# Fluoride levels in UK infant milks

**DOI:** 10.1007/s40368-016-0226-z

**Published:** 2016-05-04

**Authors:** R. M. Bussell, R. Nichol, K. J. Toumba

**Affiliations:** Department of Child Dental Health, School of Dentistry, University of Leeds, Clarendon Way, Leeds, LS2 9LU UK; Doncaster Community Dental Service, The Flying Scotsman Centre, St Sepulchre Gate West, Doncaster, DN1 3AP UK

**Keywords:** Fluoride, Infant formula, Ready-to-feed, Milk

## Abstract

**Aim:**

To provide a comprehensive report of fluoride concentration in UK infant milks and estimate their contribution to daily fluoride intake.

**Methods:**

A total of 60 formula milk products available commercially or within a hospital environment were analysed, along with eight pasteurised cow’s milk samples. Formula milk products requiring preparation were reconstituted with fresh Leeds tap water (0.02 ppmF). Fluoride concentration was measured for all products directly using an ion selective electrode after addition of low-level total ionic strength adjustment buffer.

**Results:**

The overall median fluoride concentration for the 68 infant milks was 0.025 ppmF, with a wide range of 0.002–0.282 ppmF. Analysis revealed variation between composition and manufacturer, in addition to differences between measured and labeled fluoride concentration. Although all products contained low fluoride concentration, the fluoride concentrations for formula milks used within the hospital setting (0.029 ppmF) were statistically higher in comparison to the commercial formula milk (0.016 ppmF) and cow’s milk (0.017 ppmF) products. The daily fluoride contribution from infant milks was low; 0.0034 mgF/kg body weight per day was estimated between birth and 6 months of age, further decreasing as intake of infant milk decreased with age.

**Conclusions:**

Fluoride concentration of the analysed infant milks is low, providing minimal contribution towards total daily F intake and alone are unlikely to pose a threat for the development of dental fluorosis.

## Introduction

The benefits of fluoride (F) are long established and continue to be an important component of preventive programmes aimed at eliminating the progression of dental caries. For some time it has been recognised that topical, rather than systemic exposure of F controls carious lesion development (ten Cate 1990). Adversely F has the potential, as for virtually all ingested substances, to produce undesirable effects including dental fluorosis. Although the mechanism of dental fluorosis is unclear, it is without question that it is dependent on F intake during tooth development. To minimise the risk of dental fluorosis development, monitoring F intake of children up to 3 years of age has been suggested (Buzalaf and Levy 2011), while Evans and Stamm (1991) have defined the most influential period for adverse F effects on the maxillary permanent central incisors as a 4-month period commencing around 22 months of age.

Children receive F from multiple sources and there is substantial variation among individuals (Levy et al. 1995). F levels in water is well recognised to be directly associated with severity of dental fluorosis, but the rising prevalence in non-fluoridated communities has raised questions of other potential F sources. The *Iowa Fluoride Study* (USA) has proposed sources of F including formula milk, the use of fluoridated dental products and F ingested unintentionally through the halo-effect (Levy et al. 2010). A positive association between formula milk feeding and prevalence of fluorosis at a detectable level has been reported (Do et al. 2012).

Despite recommendations of exclusively breastfeeding for the first 6 months of life (WHO 2003) and suggestion that this is becoming more common practice, almost one-fifth of mothers in the UK report to solely formula feed their baby from birth (McAndrew et al. 2012). Although powdered milks dominate the commercial market ready-to-feed (RTF) products are becoming more popular, likely due to their convenience. From 12 months of age the use of whole cow’s milk is suitable as an infant’s source of milk intake, with the introduction of semi-skimmed from 24 months (NHS 2012a). Specialised infant formula milks are available in the UK for infants with specific medical needs either on prescription or used within the hospital environment.

Numerous studies considering F concentration in formula milks have been published worldwide. UK studies have reported low F concentration in RTF formulae and powdered formula milks when reconstituted with non-fluoridated water or distilled water (Howat and Nunn 1981; Vlachou et al. 1992; Maguire et al. 2012; Zohoori et al. 2012). Wide variation has been published for F concentration of human breast milk as well as cow’s milk, but both contain F at low concentrations, reportedly up to 0.02 and 0.10 ppmF, respectively (Koparal et al. 2000).

A limitation of the published studies considering F concentration of infant milks has been related to their low sample sizes and restriction to commercial products only. In light of this, the aim of this study was to provide a current comprehensive report of the F concentration in UK infant milks and estimate their contribution to daily F intake. The study sample encompassed a wide range of infant-suitable milks including commercial formula milk products supplemented with hospital-use formula milks and fresh pasteurised cow’s milk.

## Materials and method

Formula infant milks (60) were analysed including 47 commercial and 13 hospital-use infant milks (Table 1). All commercial powdered and RTF formula milks available from the top four UK supermarkets (Kantar Worldpanel 2012) and well-known UK high street chemist were purchased, providing a sample representative of the UK market. Hospital-use formula milks provided to full-term in-patients at Leeds General Infirmary were collected. In addition, 8 fresh cow’s milk products (whole and semi-skimmed pasteurised cow’s milk samples, Table 2), suitable for infants, were bought from the top four UK supermarkets in Leeds for analysis to complete the comprehensive sample of infant milks. All products were sourced between November 2012 and February 2013.

**Table 1:** Fluoride concentration (ppmF) of commercial UK infant formula milks categorised according to the manufacturer target age and formula composition

Target age	Composition	Product name	Fluoride concentration (ppmF)	
			Powder	RTF
Commercial infant milks				
From birth	First (infant) formula^a^	Aptamil™ First	0.013	0.009
		Cow & Gate^®^ First	0.010	0.013
		HIPP Organic First	0.028	0.008
		SMA First	0.018	0.017
	Infant formula marketed for hungrier babies^a^	Aptamil™ Hungry	0.017	0.012
		Cow & Gate^®^ Hungrier baby	0.024	0.011
		HIPP Organic Hungry	0.021	0.011
		SMA Extra Hungry	0.018	0.022
	Partially hydrolysate formula^a^	Aptamil™ Comfort	0.028	–
		Cow & Gate^®^ Comfort	0.024	–
		SMA Comfort	0.008	–
	Soy protein based formula^b^	Cow & Gate^®^ Infasoy	0.025	–
		SMA Wysoy	0.002	–
	Thickened formula^a^	Aptamil™ Anti-reflux	0.014	–
		Cow & Gate^®^ Anti-reflux	0.011	–
		SMA Staydown	0.015	–
	Lactose-free formula^a^	SMA LF	0.016	–
From 6 months	Follow-on formula^a^	Aptamil™ Follow-on	0.017	0.009
		Cow & Gate^®^ Follow-on	0.023	0.009
		HIPP Organic Follow-on	0.034	0.042
		SMA Follow-on Milk	0.016	0.027
	Goodnight milk^a^	HIPP Organic Good Night Milk	0.029	–
From 12 months	Soy protein based formula^b^	Alpro Soya	–	0.282
	Growing-up milks^a^	Aptamil™ Growing-up 1 year +	0.004	0.010
		Cow & Gate^®^ Growing-up 1–2 years	0.010	0.020
		HIPP Organic Growing-up 12 m +	0.037	0.031
		SMA Toddler 1–3 years	0.014	0.015
From 24 months	Growing-up milks^a^	Aptamil™ Growing-up 2 year +	0.005	0.020
		Cow & Gate^®^ Growing-up 2–3 years	0.014	0.016
Hospital-use infant milk				
From birth	Extensively hydrolysate formula^a^	Cow & Gate^®^ Pepti-junior	0.022	–
		Nutramigen Lipil 1	0.043	–
	Thickened formula^a^	Enfamil AR	0.061	–
	Lactose-free formula^a^	Enfamil O-Lac	0.020	–
	MCT enhanced formula^c^	SHS MCT Peptide	0.008	–
		SHS Caprilon	0.031	–
		SHS Monogen	0.029	–
	Amino-acid based formula^d^	Neocate LCP	0.023	–
	High Energy RTF^a^	SMA High Energy	–	0.011
From 12 months	Thickened formula^a^	SHS Ketocal	0.099	–
		SHS Generaid plus	0.044	–
	MCT enhanced formula^c^	SHS MCT Peptide 1+	0.024	–
	Amino-acid based formula^d^	Neocate advance	0.037	–

*RTF* ready to feed

^a^Cows milk protein based

^b^Soya protein based

^c^Non-milk derived, modified fat

^d^Amino-acid based

**Table 2:** Fluoride concentration (ppmF) of fresh UK pasteurised infant milk categorised by target age

Target age	Supermarket own Products	Fluoride concentration (ppmF)
From 12 months	Asda whole milk	0.013
	Morrisons whole milk	0.019
	Sainsbury’s whole milk	0.019
	Tesco whole milk	0.016
From 24 months	Asda semi-skimmed milk	0.016
	Morrisons semi-skimmed milk	0.025
	Sainsbury’s semi-skimmed milk	0.015
	Tesco semi-skimmed milk	0.021

Preliminary studies of F concentration and potential relationships with infant milk temperature and mixing method of reconstituted powdered infant formula were conducted. No statistical difference in F concentration was observed for either potential association (*p* > 0.05). As such, reconstitution of powdered products was carried out using fresh-boiled Leeds tap water and mixed with a FISON Whirlimixer^®^ for thorough, even mixing. Powdered formulas were prepared as per the National Health Service (NHS) guidance (NHS 2012a) and the manufacturer’s instructions. F concentrations were measured within 10 min of reconstitution, while RTF products and cow’s milk were measured un-diluted at room temperature.

F concentration was measured directly, after addition of total ionic strength adjustment buffer (TISAB), using a F ion-selective electrode (Orion^®^ 9609BN) and analyser (Orion^®^ 920A). Three samples of each infant milk product were measured in duplicate, to ensure accuracy, and the mean F concentration was recorded in Microsoft Excel, 2010. Re-analysis of a randomly selected 10 % of infant milks assessed reproducibility of the method, while accuracy of the electrode was verified through analysis of an internal standard (5.0 ppmF) measured pre-, mid- and post-laboratory sessions.

Descriptive and statistical analysis was completed using SPSS v20 considering the F concentration of infant milks categorised into three groups (Table 3) and further considered target age, manufacturer (supermarket for cow’s milk) and composition. Statistical significance was considered for *p* < 0.05.

**Table 3:** Median (range) of fluoride concentration for UK infant milks

Infant milk group	No. of samples	Fluoride concentration (ppmF) Median (range)
Commercial		
All	47	0.016 (0.002–0.282)
Powder^a^	28	0.017 (0.002–0.037)
Ready-to-feed (RTF)	19	0.015 (0.008–0.282)
Hospital-use	13	0.029 (0.007–0.099)
Fresh pasteurised cow’s milk	8	0.017 (0.012–0.025)

^a^Powdered products reconstituted with Leeds non-fluoridated tap water

### Daily intake

Infant milk contribution to daily dietary F consumption, up to 3 years, was estimated considering NHS infant milk intake recommendations [up to 6 months—200 ml/kg body weight (bw) per day (NHS 2012b); 6 months up to 12 months—600 ml per day (NHS 2008); 12 months and over—300 ml per day (NHS 2013)]. Up to 6 months of age recommended milk intake increases with growth and the UK-World Health Organisation growth charts (female 50th weight centile) for 0–4 years were utilised to calculate the milk intake. The daily F intake from infant milk consumption was calculated using the median F concentration of infant milks categorised by the manufacturer target-age. Each product was categorised once.

## Results

The median F concentration for the 68 infant milks was 0.025 (0.002–0.282) ppmF. There was a wide range in F concentration across the study sample, although the F concentration for individual products was considered low. Table 3 shows the descriptive statistical analysis for the three study groups. The highest F concentration (0.282 ppmF), by a considerable margin, was seen in the only RTF soya based commercial product within the study sample (*Alpro Soya*). Despite the wide variation of F concentration for RTF commercial products, a similar median F concentration was seen comparing commercial formula product format (powdered vs. RFT) (Table 3). Comparison of the three study groups demonstrated a statistically significant higher F concentration of hospital-use formula milk compared to commercial formula and cow’s milk products (Krusal Wallis, *p* = 0.012).

**Fig. 1:**
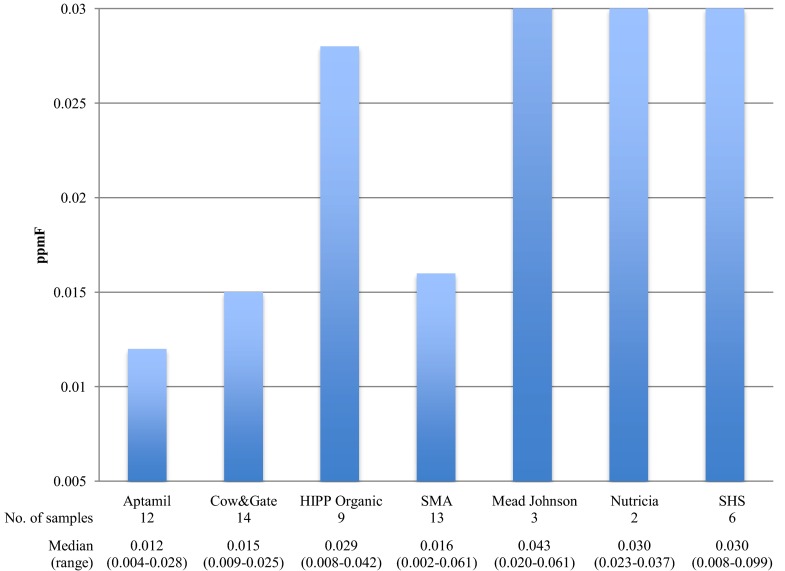
Comparison of the median fluoride concentration (ppmF) of infant formula milks categorised by manufacturer (excluding manufacturer Alpro; represented by one product (Alpro *Soya*) with a F concentration of 0.282 ppmF Krusal Wallis and Mann–Whitney *p* > 0.005 Bonferroni correction)

**Fig. 2:**
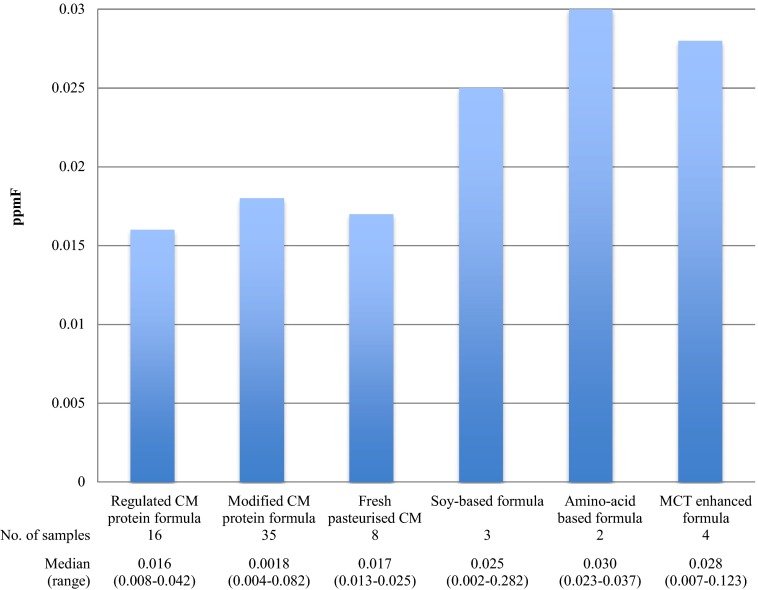
Comparison of the median fluoride concentration (ppmF) of UK infant milks categorised by product composition. *CM* cow’s milk. (Krusal Wallis, *p* = 0.328)

Comparable F concentrations were seen considering the infant milk manufacturer target age (Table 4). Figures 1 and 2 show the differences seen for comparison of the infant milks considering their manufacturer and composition respectively, with no statistical significant differences. Generally, higher median F concentrations were seen for the manufacturer’s *Mead Johnson, Nutricia and SHS*, reflective of the higher F concentration seen for hospital-use infant milks verses commercial products (Fig. 1). The manufacturer *Alpro* (excluded from Fig. 1) was represented by one product, *Alpro Soya*, and presents the greatest F concentration (0.282 ppmF) by a considerable margin. For pasteurised milk, a comparable F concentration was seen in a comparison of the supermarket products.

Considering a relationship between milk composition and F concentration, cow’s milk protein based products contained a lower median F concentrations (Fig. 2). An interesting observation was the measured F concentration, for the majority, was lower than the labeled F concentration where displayed and this difference was statistically significant (One sample *t* tests, Bonferroni correction *p* < 0.002).

**Table 4:** Median (range) of infant milks categorised by manufacturer target age and estimated daily fluoride intake

Target age	No. of samples	Fluoride concentration (ppmF)* Median (range)	Body weight^a^ (kg)	Fluoride intake^b^	
				mgF/kg bw per day	mgF per day
0–6 months	34	0.017 (0.002–0.061)	3.4–6.9	0.0034	0.008–0.018
6–12 months	9	0.023 (0.009–0.042)	7.3–8.7	≤0.002	0.014
12–36 months	25	0.018 (0.004–0.282)	8.9–13.8	<0.001	0.005

* Statistical comparison of fluoride concentration for target age, Krusal Wallis, *p* = 0.513

Each product categorised once

^a^Body weight (bw) taken from UK-WHO growth charts 0–4 years (female 50th centile)

^b^Fluoride intake calculated using NHS milk intake recommendations (NHS 2008, 2012b, 2013)

F concentration of Leeds tap water was measured, with a median F concentration of 0.019 (0.012–0.02) ppmF, confirming a low F level. Possible batch variation of the powdered formula products was considered through analysis of five different batch numbered items sourced throughout the UK, for an individual powdered commercial product [median F concentration (range) 0.005 (0.004–0.008) ppmF]. No statistical difference was observed (Krusal Wallis, *p* = 0.406).

Good reproducibly of the method was demonstrated with a percentage agreement range of 81–96 % for re-analysis of 10 % of the total study sample. Statistical analysis of an internal standard within and between the test sessions verified the accuracy of the F ion-selective electrode (F test, *p* = 0.106).

### Daily intake

Infant milks provide a small contribution to the daily F intake, ranging from 0.008 mgF at birth to 0.005 mgF at 36 months (Table 4). The daily F contribution from infant milks is greatest at 6 months of age, as recommended infant milk consumption increases, reaching a peak F intake of 0.018 mgF per day. From birth up to 6 months of age, the F intake from the appropriate age-targeted infant milks would be 0.003 mgF/kg bw per day.

## Discussion

This study provides a comprehensive analysis of the F concentration in infant milks and overall the F concentrations were low. A large sample of 47 commercial powdered and RTF products were analysed, representative of the UK market. In addition, hospital-use infant milks were included in the overall sample which have not to the authors knowledge been reported in the literature to date.

UK studies have generally reported lower F concentration for infant milks in comparison to international studies, and the present study supports this observation (Table 5). Previous studies have varied in their sample size, with generally a small number of infant milks considered. It is prudent to be mindful of the sample sizes and sample products when comparing F concentration. Published studies have shown varied methodologies for investigating F concentration of infant milks, utilising direct and indirect methods (Table 5). The method used for the current study was determined considering published studies and following preliminary investigations to eliminate confounding factors. As such the direct method was used utilising a F ion-selective electrode. A limitation of the F ion-selective electrode is the level of F detection (0.02 ppmF); however, for this study the direct method provided a measurement to a meaningful level. Good reproducibility was shown and the accuracy of the F ion-selective electrode was verified.

**Table 5:** Summary of UK and international studies on fluoride concentration in commercially available infant formula milks

Author	No. of products (format)	Fluoride concentration (ppmF)		Methodology
		Mean (median^a^)	Range	
UK studies				
Howat et al.(1981)	8 (Powder^b^)	–	0.02–0.08	F-ISE
Vlachou et al. (1992)	6 (Powder^b^) 6 (RTF)	–	0.16–0.70 0.01–0.11	MD
Maguire et al. (2012)	11 (RTF)	0.02^a^	0.01–0.03	F-ISE
Zohoori et al. (2012)	18 (Powder^b^)	0.04	0.02–0.18	MD
Present study	28 (Powder^b^) 19 (RTF)	0.02^a^ 0.02^a^	<0.01–0.04 <0.01–0.28	F-ISE
International studies				
Johnson and Bawden (1987) USA	10 (Powder^b^) 15 (RTF)	0.12 0.21	0.03–0.24 0.05–0.38	MD
Silva and Reynolds (1996) Australia	11 (Powder^b^)	0.24	0.03–0.53	MD
Cressey (2010) New Zealand	32 (Powder^b^)	0.07	0.02–0.20	MD
Nohno et al. (2011) Japan	22 (Powder^b^)	0.09	0.04–0.24	MD

*RTF* ready to feed, *F*-*ISE* fluoride ion-selective electrode (direct method), *MD* micro-diffusion (indirect method)

^a^Median fluoride concentration

^b^Powder formula prepared with distilled/deionised or non-fluoridated water

Reconstitution of powdered formula was achieved with fresh Leeds tap water. Due to the low F concentration of Leeds tap (0.02 ppmF) water, comparisons with other F studies of formula milks should focus on powdered formula reconstituted with distilled or de-ionised water of similar low F concentration (Table 5).

A study of powdered infant milks available in Leeds published by Vlachou et al. (1992), reported a higher F concentration by comparison to this present study. Importantly, the 1992 study analysed a considerably smaller sample size and no details of manufacturer were published, as such comparisons are limited. In a 2012 UK study, Zohoori and colleagues considered comparable powdered formula milks products to the current analysis, although a smaller sample size, reporting a higher median F concentration of 0.04 μg/g. Despite higher F values reported, the published evidence suggests low F concentration is found in powdered formula milks available in the UK (Table 5).

A similar median F concentration of RTF infant milks to this study has been reported in the literature (Maguire et al. 2012). Wide variation of F concentration in RTF infant milks was found in that study and this observation is also supported by previous published UK studies (Vlachou et al. 1992; Maguire et al. 2012). As there is no need for reconstitution of RTF products there are fewer factors influencing F concentration, therefore with greater control over the estimated ingested F and comparison with previous studies.

Differences between measured and labeled (where provided) F concentration was found in this study. Comparison of the manufacturers’ reported concentrations on labels and the study results are limited due to multiple influences in final F concentration e.g. place of manufacture and F level of fluid used for reconstitution. Such details are not provided on the label and manufacturers may record upper limits as a safety margin.

Compositional legislation of formula milk is restricted to infant and follow-on formulas, recommended from birth and 6 months respectively. A maximum limit for F in such formula milk has been set at 100 µg/100 ml, with no lower limit (European Commission Directive 2006/141/EC 2006). F concentration for all the formula milks analysed (measured and labeled) in this study fall well below this maximum limit.

This paper is the first to report on F concentration of hospital-used formula milk for full term infants. Variation was observed between the three study groups, and despite a statistically significant higher F concentration in the hospital-used milks this is unlikely to be clinically significant. The hospital-use infant milk group comprised a local convenience sample, which included a range of compositions and manufacturers. Variation in F concentrations was observed within the hospital-use infant milk group but no significant differences were found considering target age, manufacturer or composition. Although the NHS recommended infant milk intake levels (NHS 2008, 2012b, 2013) are taken into consideration, an individual formula milk plan is determined. It is therefore impossible to accurately determine the daily intake of F for in-patients consuming hospital-use formula milks due to the great variability in feeding regime. Despite this, due to the comparatively low F level in the hospital-use infant milks, total daily F intake levels from hospital-use formula milks are likely to be low.

The F concentration in fresh cow’s milk available in local supermarkets had a similar value to the formula infant milks analysed. Variation in F concentration of cow’s milk has been published, however the results of this present study fall within this reported range (Dirks et al. 1974; Koparal et al. 2000). A study considering an extensive number of dairy milks purchased from supermarkets in the USA reported an average similar to the present study (Liu et al. 1995). Research has suggested the F concentration of cow’s milk is influenced by F ingestion (Dirks et al. 1974). The cow’s milk analysed in this present study was purchased in Leeds, a non-fluoridated region and the F concentration was comparable to Leeds tap water. Fluoride levels in water varies throughout the UK and this may be reflected in the F levels in regional fresh cow’s milk.

### Daily intake

Daily F intake estimations from infant milks analysed in this study supports existing evidence that infant milks alone contribute only marginally to the total daily F intake. Up to 6 months of age the estimated F intake from infant milks reported herein provided 0.003 mgF/kg bw per day, similar to a suggestion by Maguire et al. (2012) of 0.002 mgF/kg bw per day.

The precise level of F exposure required to cause dental fluorosis is unknown, but there has been general agreement that the total daily F intake should not exceed 0.1 mgF/kg bw per day (Burt 1992). Overall the levels of F intake from infant milks in this study fell well below this recommendation and specifically considering the risk period for the permanent central incisors (Evans and Stamm 1991), the estimated daily F intake should be <0.001 mgF/kg bw per day between 22 and 26 months of age. Previous research has suggested associations between F concentration of formula milks and dental fluorosis, although this association was dependent on the level of F in the water supply (Berg et al. 2011).

The results of low F concentration of formula milks requiring reconstitution (with non-fluoridated tap water) in this present study supports previous conclusions that F intake is highly dependable on the water F concentration used for reconstitution rather than the F concentration of the powdered formula itself (Cressey 2010; Zohoori et al. 2012). A study using fluoridated water (0.9ppmF) for reconstitution demonstrated a median F concentration for 18 formula milks (0.92ppmF) comparable to the F water level used (Zohoori et al. 2012). Fluoridated water used following the NHS milk intake levels (NHS 2008, 2012b, 2013) would lead to intake levels of 0.180, <0.070 and ≤0.030 mgF/kg bw per day for infants up to 6, 6–12 and 12 months onwards respectively. Raising concern, in particular for infants up to 6 months of age. The American Academy of Pediatric Dentistry has provided guidance recommending the use of RTF products in regions where dental fluorosis is of concern due to high water F levels (Berg et al. 2011).

A recent study comparing F intake between fluoridated and non-fluoridated regions of North England reported a mean total daily F intake for infants up to 12 months of age at 0.107 and 0.024 mgF/kg bw per day respectively (Zohoori et al. 2014). This suggests the water F content has a major influence on the total daily F intake for infants. Estimating total daily F intake can be challenging and it is important to understand the various sources and factors influencing the overall amount (Maguire and Zohoori 2013).

## Conclusions

Fluoride concentration of analysed UK infant milks is low, providing minimal contribution towards total daily F intake and alone are unlikely to pose a threat for the development of dental fluorosis.

## References

[CR1] Berg J, Gerweck C, Hujoel PP (2011). Evidence-based clinical recommendations regarding fluoride intake from reconstituted infant formula and enamel fluorosis: a report of the American Dental Association Council on Scientific Affairs. J Am Dent Assoc.

[CR2] Burt BA (1992). The changing patterns of systemic fluoride intake. J Dent Res.

[CR3] Buzalaf MA, Levy SM (2011). fluoride intake of children: considerations for dental caries and dental fluorosis. Monogr Oral Sci.

[CR4] Cressey P (2010). Dietary fluoride intake for fully formula-fed infants in New Zealand: impact of formula and water fluoride. J Public Health Dent.

[CR5] Dirks OB, Jongeling-Eijndhoven JM, Flissebaalje TD, Gedalia I (1974). Total and free ionic fluoride in human and cow’s milk as determined by gas-liquid chromatography and the fluoride electrode. Caries Res.

[CR6] Do LG, Levy SM, Spencer AJ (2012). Association between infant formula feeding and dental fluorosis and caries in Australian children. J Public Health Dent.

[CR7] European Commission Directive 2006/141/EC. 2006. http://eur-lex.europa.eu/LexUriServ/LexUriServ.do?uri=OJ:L:2006:401:0001:0033:EN:PDF. Accessed 4th May 2013.

[CR8] Evans RW, Stamm JW (1991). An epidemiologic estimate of the critical period during which human maxillary central incisors are most susceptible to fluorosis. J Public Health Dent.

[CR10] Howat AP, Nunn JH (1981). Fluoride levels in milk formulations. Supplementation for infants. Br Dent J.

[CR11] Johnson J, Bawden JW (1987). The fluoride content of infant formulas. Pediatr Dent.

[CR12] Kantar Worldpanel. 2012. http://www.kantar.com/consumer/shoppers/080712/. Accessed 14th July 2013.

[CR13] Koparal E, Ertugrul F, Oztekin K (2000). Fluoride levels in breast milk and infant foods. J Clin Pediatr Dent.

[CR14] Levy SM, Kiritsy MC, Warren JJ (1995). Sources of fluoride intake in children. J Public Health Dent.

[CR15] Levy SM, Broffitt B, Marshall TA, Eichenberger-Gilmore JM, Warren JJ (2010). Associations between fluorosis of permanent incisors and fluoride intake from infant formula, other dietary sources and dentifrice during early childhood. J Am Dental Assoc.

[CR16] Liu C, Wyborny LE, Chan JT (1995). Fluoride content of dairy milk from supermarket; a possible contributing factor to dental fluorosis. Fluoride..

[CR17] McAndrew F, Thompson J, Fellow L, et al. Infant Feeding Survey 2010: Summary. Health and Social Care Information Centre, IFF Research 2012. http://www.hscic.gov.uk/catalogue/PUB08694/Infant-Feeding-Survey-2010-Consolidated-Report.pdf.

[CR18] Maguire A, Omid N, Abuhaloob L, Moynihan PJ, Zohoori FV (2012). Fluoride content of ready-to-feed (RTF) infant food and drinks in the UK. Community Dent Oral Epidemiol.

[CR19] Maguire A, Zohoori FV (2013). Fluoride balance in infants and young children in the UK and its clinical relevance for the dental team. Br Dent J.

[CR20] NHS. Weaning: starting sold food. 2008. http://webarchive.nationalarchives.gov.uk/20130107105354/, http://www.dh.gov.uk/prod_consum_dh/groups/dh_digitalassets/documents/digitalasset/dh_084164.pdf. Accessed 11th May 2013.

[CR21] NHS. Making up infant formula. 2012a. http://www.nhs.uk/conditions/pregnancy-and-baby/pages/making-up-infant-formula.aspx#close. Accessed 4th Aug 2012.

[CR22] NHS. Guide to bottle feeding. 2012b. http://www.nhs.uk/start4life/documents/pdfs/start4life_guide_to_bottle_feeding.pdf. Accessed 4th May 2013.

[CR23] NHS. Milk and dairy foods. 2013. http://www.nhs.uk/Livewell/Goodfood/Pages/milk-dairy-foods.aspx. Accessed 16th March 2013.

[CR24] Nohno K, Zohoori FV, Maguire A (2011). Fluoride intake of japanese infants from infant milk formula. Caries Res.

[CR25] Silva M, Reynolds EC (1996). Fluoride content in infant formula in Australia. Aust Dent J.

[CR26] ten Cate JM. In vitro studies of the effects of fluoride on de- and remineralisation. J Dent Res. 1990;69. doi:10.1177/00220345900690S120. 10.1177/00220345900690S120 2179322

[CR27] Vlachou A, Drummond BK, Curzon ME (1992). Fluoride concentrations of infant foods and drinks in the United Kingdom. Caries Res.

[CR28] World Health Organization. The World Oral Health Report. 2003. http://www.who.int/oral_health/media/en/orh_report03_en.pdf. Accessed 14th March 2012.

[CR29] Zohoori FV, Moynihan PJ, Omid N, Abuhaloob L, Maguire A (2012). Impact of water fluoride concentration on the fluoirde content of infant foods and drinks requiring preparation with liquids before feeding. Community Dent Oral Epidemiol.

[CR30] Zohoori FV, Whaley G, Moynihan PJ, Maguire A (2014). Fluoride intake of infants living in non-fluoridated and fluoridated areas. Br Dent J.

